# Immunoinformatic Docking Approach for the Analysis of KIR3DL1/HLA-B Interaction

**DOI:** 10.1155/2013/283805

**Published:** 2013-08-01

**Authors:** Alba Grifoni, Carla Montesano, Atanas Patronov, Vittorio Colizzi, Massimo Amicosante

**Affiliations:** ^1^Department of Biology, University of Rome “Tor Vergata”, Via della Ricerca Scientifica 1, 00133 Rome, Italy; ^2^ProxAgen Ltd., blv. Sitnyakovo 35, 1505 Sofia, Bulgaria; ^3^Department of Biomedicine and Prevention, University of Rome “Tor Vergata”, Via Montpellier 1, 00133 Rome, Italy

## Abstract

KIR3DL1 is among the most interesting receptors studied, within the killer immunoglobulin receptor (KIR) family. Human leukocyte antigen (HLA) class I Bw4 epitope inhibits strongly Natural Killer (NK) cell's activity through interaction with KIR3DL1 receptor, while Bw6 generally does not. This interaction has been indicated to play an important role in the immune control of different viral infectious diseases. However, the structural interaction between the KIR3DL1 receptor and different HLA-B alleles has been scarcely studied. To understand the complexity of KIR3DL1-HLA-B interaction, HLA-B alleles carrying Bw4/Bw6 epitope and KIR3DL1∗001 allele in presence of different peptides has been evaluated by using a structural immunoinformatic approach. Different energy minimization force fields (ff) have been tested and NOVA ff enables the successful prediction of ligand-receptor interaction. HLA-B alleles carrying Bw4 epitope present the highest capability of interaction with KIR3DL1∗001 compared to the HLA-B alleles presenting Bw6. The presence of the epitope Bw4 determines a conformational change which leads to a stronger interaction between nonpolymorphic arginine at position 79 of HLA-B and KIR3DL1∗001 136–142 loop. The data shed new light on the modalities of KIR3DL1 interaction with HLA-B alleles essential for the modulation of NK immune-mediated response.

## 1. Background

Killer immunoglobulin receptor (KIR)3DL1 is among the most interesting receptors studied within the KIR family. It interacts with polymorphisms located on human leukocyte antigen (HLA)-A and HLA-B molecules that have been widely associated with the control of several infections and autoimmune disorders [1–6]. Interestingly, not only the HLA-B molecule *per se* is involved in the inhibition of natural killer (NK) cell cytotoxic activity, but a critical role is also played by the peptide loaded in the HLA-B-binding pocket [7]. In particular, the relative positions 7 and 8 of the peptide bound to HLA class I are critical for the recognition since they directly interact with amino acids from HLA-B at positions 77, 80, 81, 82, and 83 which form Bw4/Bw6 epitope [7, 8]. Bw4 epitope (Asn 77, Ile 80, Ala 81, Leu 82, and Arg 83) has been associated with a strong inhibition of NK cell's activity through KIR3DL1 receptor interaction, while Bw6 epitope (Ser 77, Asn 80, Leu 81, Arg 82, and Gly 83) does not interact with KIR3DL1 [3, 9, 10], though a weak bound with one of the alleles has been described [11]. 

The KIRs/HLA-B complex crystal structure has been resolved and deposited in the Protein Data Bank [12]. In particular, KIR3DL1 D1 domain is capable to interact both with the HLA bond peptide and the HLA class I molecule alpha-1 domain including the Bw4/Bw6 epitope [12]. This notion supports the observed differences in NK mediated cytotoxic effects due to peptides with different amino acids residues in the relative positions P7 and P8 [2, 8, 13, 14]. 

The basis for this effect might be due to different, not mutually exclusive mechanisms: (i) direct steric effect, (ii) lack of complementarities between the peptide and KIR3DL1∗001, and (iii) the induced conformational change in the Bw4 epitope [12]. 

To assess the complexity of KIR3DL1-HLA-B interaction and its role in the immune regulation we studied the formation of complexes between different HLA-B alleles carrying Bw4/Bw6 and KIR3DL1∗001 allele in presence of different peptides using a structural immunoinformatic approach.

## 2. Material and Methods

Seven HLA-B alleles have been evaluated according to carrying Bw4/Bw6 epitope supertype [15] and allele frequency in different human populations [16] (Table 1). Quality of the models based on X-ray crystallography experiments or homology modeling in SWISS model workspace [17] has been evaluated with PROSESS server [18]. In order to compare different HLA-B alleles, peptides with the similar EC_50_ (200 nM) were selected as references from IEDB database [19]. In the analysis of HLA-B alleles belonging to the same supertype, the same reference bound peptide has been used in all evaluated HLA-B molecules, even if presenting a different EC_50_ for the tested alleles.

Homology modeling has been applied using HLA-B57_KIR3DL1 complex as a template (PDB ID: 3VH8). A MOTIF alignment [20] of different HLA-B structures with HLA-B57 followed by HLA-B replacement has been performed. The different HLA-bound peptides have been generated through single-point amino acid substitution. 

All models were subjected to energy minimization by using Yet Another Scientific Artificial Reality Application, (“YASARA” http://www.yasara.com/) and the implemented AMBER and NOVA force fields (ff). The same minimization protocol was used with both force fields. This is represented by a combination of steepest descent performed at the beginning and followed up by a simulated annealing. The calculation is performed until the convergence criteria are met or the maximum number of steps is exceeded.

To study differences between AMBER and NOVA ff and to prevent bias due to a low number of data points, HLA-B alleles analyzed have at least 100 data points for both binders (EC_50_ < 500 nm) and nonbinders (EC_50_ > 20000 nm) peptides in the IEDB database (HLA-B∗35:01: binders = 162, non-binders = 108; HLA-B∗51:01: binders = 142, non-binders = 220; HLA-B∗57:01: binders = 144, non-binders = 205; HLA-B∗58:01: binders = 154, non-binders = 516) [19].

HLA-B_KIR3DL1 complexes were therefore analyzed using NOVA ff for minimization followed by AMBER ff for energy calculation as previously described [21]. 

The binding free energy (Δ*G*) has been calculated as the sum of the interactive and solvation energies. KIR3DL1 binding free energy has been calculated as the difference between KIR3DL1-HLA-B-peptide complex and the sum of HLA-peptide complex and KIR3DL1 alone as previously reported [22].

The difference in the KIR3DL1 binding free energy (ΔΔ*G*) in presence of different HLA bound peptides has been calculated as differences between the Δ*G* of the KIR3DL1/HLA-peptide-(substituted) complex and the KIR3DL1/HLA-peptide-(reference) complex as previously reported [23]. The more negative is the value the stronger is the predicted KIR3DL1 interaction. 

Statistical analysis has been performed using the paired nonparametric Wilcoxon test, on GraphPad Prism 5.0 (San Diego, CA, USA). Graphics are represented as whiskers of 10–90 percentiles of the distribution obtained for the 180 single-amino acid substitutions in the bound peptide to HLA-B allele. 

## 3. Results and Discussion

### 3.1. Immunoinformatic Approach

To select the best strategy for evaluation of the HLA-peptide interaction with KIR3DL1 receptor, different energy minimization ff have been tested in parallel. Among them, AMBER and NOVA ff are the most used in bioinformatics approach [24, 25]. To compare the applicability on our system of the two ff, amino acid substitution in the HLA bound peptide has been performed according to a list of binding (EC_50_ < 500 nM) and nonbinding peptides (EC_50_ > 20000 nM) from IEDB. NOVA ff demonstrated better performance compared to AMBER for distinguishing the binding and nonbinding peptides (HLA-B NOVA ff: mean value binders −129.6 ± 198 kj/mol, mean value nonbinders −399.6 ± 181.8 kj/mol, *P* = 0.0286; HLA-B AMBER ff: mean value binders −637.6 ± 154.9 kj/mol, mean value nonbinders −929.4 ± 423.8, *P* = 0.1714).

The use of AMBER allowed a greater distortion than NOVA in the cocrystallization structure of HLA-B∗57:01-KIR3DL1 when used for energy minimization (AMBER minimized structure RMSD = 0.725; NOVA minimized structure RMSD = 0.573). This is in agreement with previous observation of other molecular systems comparing the two ff [24]. Therefore, we considered NOVA ff for the further study, since it was capable of predicting successfully the ligand-receptor interaction due to the lower molecular distortion caused on the entire complex.

### 3.2. Role of HLA Bw4/Bw6 Epitope in KIR3DL1∗001 Interaction

To evaluate the contribution of Bw4 and Bw6 epitopes together with the peptide substitution in KIR3DL1/HLA interaction, different HLA-B alleles carrying Bw4 or Bw6 epitope have been analyzed (Table 1). 

**Figure 1:**
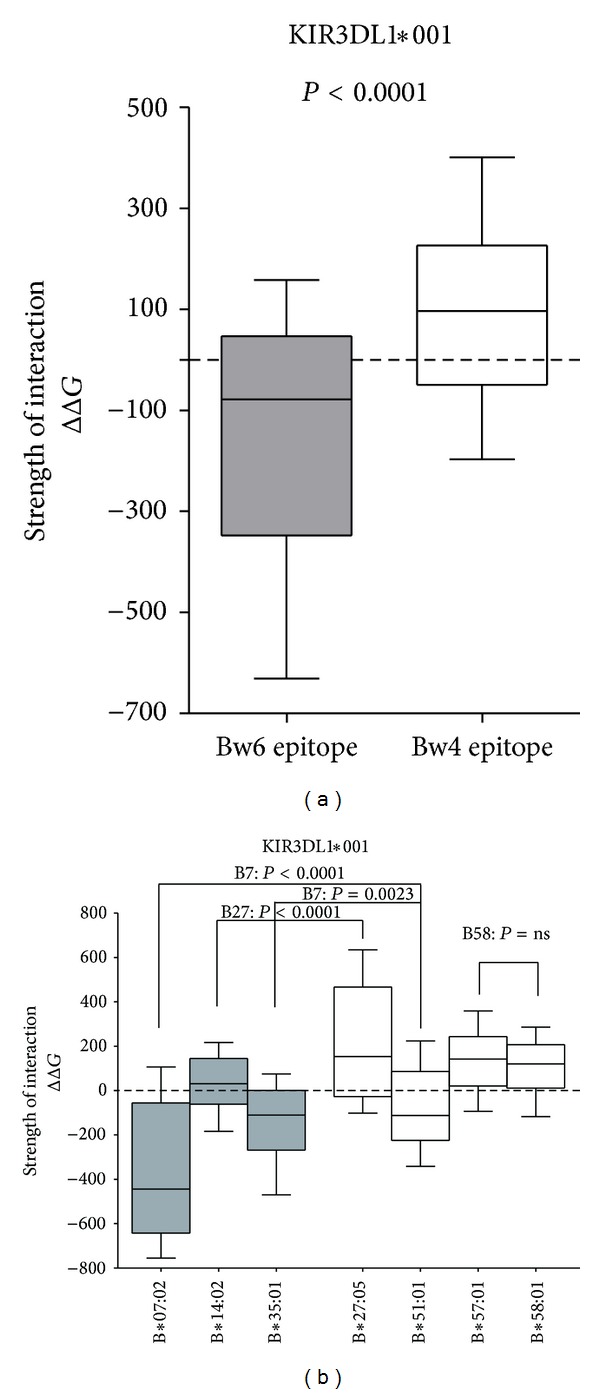
Contribution of epitope Bw4/Bw6 in KIR3DL1 interaction. Here is shown the strength of interaction between HLA-B alleles carrying epitope Bw4 and Bw6 as the difference in the estimated binding free energy (ΔΔ*G*) between the substituted peptide and the reference. The higher is the value the stronger is the interaction. (a) Overall interaction between HLA-B alleles grouped based on carrying epitope Bw4 or Bw6. (b) Interaction of each HLA-B allele with KIR3DL1∗001: the comparison between HLA-B alleles belonging to the same supertype has been performed. Each HLA-B distribution is composed by 180 points corresponding to each complex with a single-peptide amino acid substitution and is reported as 10–90 percentile whiskers. All the statistical analysis has been performed using paired, nonparametric, Wilcoxon test.

The presence of epitope Bw4 determines a higher strength of interaction with KIR3DL1∗001 allele respect to epitope Bw6 (*P* < 0.0001, Figure 1(a)). This is in agreement with previous experimental and association studies showing a strongest interaction of Bw4 epitope with KIR3DL1 respect to Bw6 [3, 9, 10]. 

The distribution of the ΔΔ*G* for all the substituted peptides for each HLA-B allele has been further analyzed. HLA-B∗07:02 has the weakest interaction with KIR3DL1∗001 followed by HLA-B∗35:01 as previously reported in experimental data obtained through flow cytometry and cytotoxic activity assay [26–28]. On the contrary, HLA-B∗27:05 has the strongest interaction with KIR3DL1∗001 followed by HLA-B∗57:01 and HLA-B∗58:01 (Figure 1(b)). The KIR3DL1 interaction capability of these three HLA-B alleles has been independently observed and confirmed in previous literature data mostly through cytotoxic assay [1, 10, 11, 26, 29, 30]. 

Worth-noting, among HLA-B alleles carrying Bw6 epitope, HLA-B∗14:02 has the highest interaction with KIR3DL1∗001. However, it presents a weaker interaction compared to the Bw4 allele HLA-B∗27:05, the other allele belonging to the B27 supertype (*P* < 0.0001; Figure 1(b)). 

On the contrary, among HLA-B alleles carrying Bw4 epitope, HLA-B∗51:01 has the weakest interaction with KIR3DL1∗001. However, the interaction is stronger respect to Bw6 alleles HLA-B∗07:02 and HLA-B∗35:01, all belonging to the B7 supertype (B∗07:02 versus B∗51:01, *P* < 0.0001; B∗35:01 versus B∗51:01, *P* = 0.0023; Figure 1(b)). 

Finally, no difference in KIR3DL1∗001 interaction has been observed between HLA-B∗57:01 and HLA-B∗58:01 (*P* = 0.1022; Figure 1(b)) belonging to B58 supertype both carrying Bw4 epitope. 

### 3.3. Role of Arg79 in KIR3DL1/HLA-B Interaction

**Figure 2:**
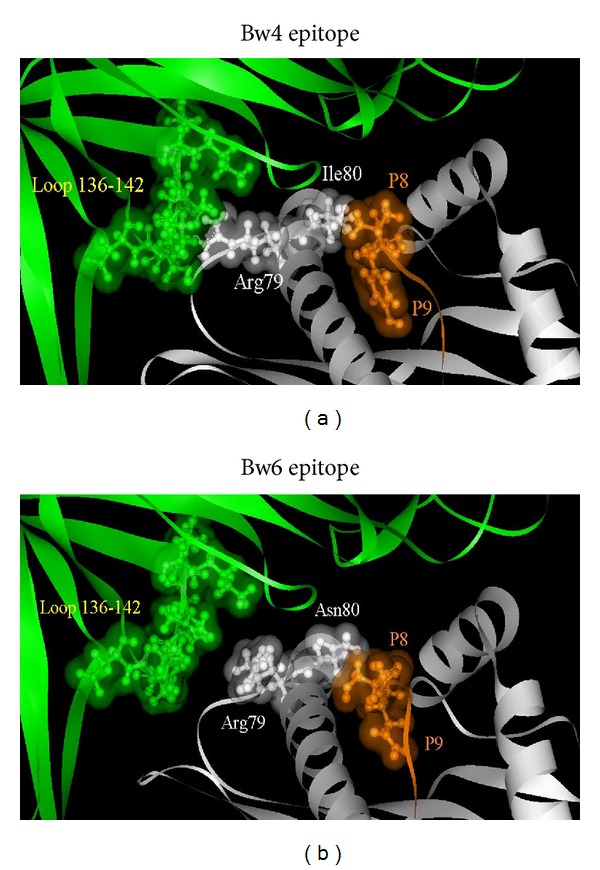
HLA-B and KIR3DL1∗001 interaction binding site. Here is shown the interaction of KIR3DL1∗001 with HLA-B and the most significant differences in the contact surface between Bw4 and Bw6 epitope ((a) and (b) resp.). In green KIR3DL1, in grey HLA-B and in orange the peptide are shown. For each amino acid residue in the site of interaction Van Deer Walls surface was calculated and amino acid residues responsible for the contact surface are named and shown with the parent color as ball sticks.

The contact residues in the HLA-B and KIR3DL1∗001 interaction have been investigated in the different HLA-B alleles carrying Bw4 and Bw6 epitope. The main difference has been observed in amino acid residues 136–142 of D1 domain of KIR3DL1∗001 interacting with the nonpolymorphic arginine 79 of the HLA-B (Figure 2). In presence of Bw4 epitope (Figure 2(a)), Arg79 is directed towards the 136–142 loop, while in presence of Bw6 epitope Arg79 changes the conformation (Figure 2(b)). This represents the most critical structural aspect at the basis of the Bw4/Bw6 different interaction capability of KIR3DL1 with HLA-B molecule.

## 4. Conclusions

Here we confirm for the first time via structural immunoinformatic approach, the highest interaction of HLA-B alleles carrying Bw4 epitope with KIR3DL1∗001 compared to epitope Bw6. The presence of epitope Bw4 is determining a conformational change which strengthens the interaction between the nonpolymorphic HLA-B Arg79 residue and KIR3DL1∗001 136–142 loop. In conclusion, the study of KIR3DL1-HLA-B aims to explain the mechanisms of immune modulation of NK cells immune response and may provide the basis for the design of more specific immunotherapeutic approaches in infectious diseases and immune disorders.

## Figures and Tables

**Table 1:** List of HLA-B alleles selected for immune-informatics approach. Alleles were selected depending on Bw4/Bw6 epitope, supertype [15], allele frequency in different population (the observed range of frequency among all the human populations is indicated) [16], and presence of crystallization structure in PDB databank. For each HLA-B supertype, using IEDB database a binding peptide with a similar EC_50_ value (*≈*200 nM) was selected.

HLA	Epitope	Supertype	Allele frequency	Peptide	PDB code
				EC_50_ *≈* 200 nM	
B∗07:02	Bw6	B7	0–0.1	MPVGGQSSF	
B∗14:02	Bw6	B27	0–0.07	MPAYIRNTL	
B∗35:01	Bw6	B7	0–0.1	MPVGGQSSF	3LKN
					
B∗27:05	Bw4	B27	0–0.2	MPAYIRNTL	3BP4
B∗51:01	Bw4	B7	0–0.08	MPVGGQSSF	
B∗57:01	Bw4	B58	0–0.06	YTAVVPLVY	3VH8
B∗58:01	Bw4	B58	0–0.1	YTAVVPLVY	
